# The role of general practitioners in Reunion in detecting alcohol use in pregnant women and identifying fetal alcohol spectrum disorder: a qualitative study

**DOI:** 10.1186/s13690-023-01221-0

**Published:** 2023-12-06

**Authors:** Sébastien Leruste, Louise Delfarguiel, Bérénice Doray, Coralie Loubaresse, Laetitia Sennsfelder, Thierry Maillard, Catherine Marimoutou, Michel Spodenkiewicz

**Affiliations:** 1 UFR Santé, University of La Réunion, 97410 Saint-Pierre, France; 2 INSERM CIC 1410, CHU de La Réunion, Saint-Pierre, France; 3 Service de Génétique - CHU de La Réunion, Saint-Denis, France; 4 Laboratoire EPI, Université & CHU de La Réunion, Saint-Denis, France; 5 Centre Ressources TSAF - Fondation Père Favron - CHU de La Réunion, Saint-Pierre, France; 6 SAF-Ocean India, Saint-Louis, France; 7 Moods Team, INSERM UMR-1178, CESP, Le Kremlin-Bicêtre, France; 8 McGill Group for Suicide Studies, Douglas Mental Health University Institute, Department of Psychiatry, McGill University, Montréal, Canada

**Keywords:** Fetal alcohol spectrum disorders, General practitioners, Qualitative research

## Abstract

**Background:**

Fetal Alcohol Spectrum Disorder (FASD) is the leading cause of non-genetic intellectual disability and social maladjustment in children. International guidelines recommend abstinence from alcohol during pregnancy. Réunion is the most affected of all French regions with an estimated Fetal Alcohol Spectrum (FAS) prevalence of 1.2‰ births. General practitioners (GPs) are at the forefront of identifying patients with FASD. Objective: To understand how GPs identify FASD.

**Methods:**

Qualitative study using a grounded theory approach, through semi-structured face-to-face interviews with GPs. Interviews were conducted with the aim of reaching theoretical saturation. These were transcribed verbatim and then analyzed by four researchers to ensure triangulation of the data.

**Results:**

GPs reported barriers to the identification of FASD: challenges in overcoming social taboos and paradoxical injunctions, the influence of limited knowledge and experience, non-specific and highly variable symptoms, ambiguous classification and method of diagnosis involving the mobilization of a multidisciplinary team and lengthy consultations. Conversely, they felt competent to identify neurodevelopmental disorders of any cause, but were concerned about the long waiting time to access specialized care. From the perspective of GPs, it is crucial to prioritize promotion and training aimed at improving the identification and coordination of care pathways for children diagnosed with neurodevelopmental disorders, such as FASD.

**Supplementary Information:**

The online version contains supplementary material available at 10.1186/s13690-023-01221-0.

**Table:** 

Text box 1. Contributions to the literature	
- Exploring GPs’ representations of the identification of FASD in the general population is a novel approach to the scientific literature, which is more interested in targeted populations, such as Canadian and Australian Aboriginal people, or specialist care. - The barriers and motivations faced by general practitioners in identifying FASD will enable decision makers and institutions to consider the resources needed to improve identification. - The findings help to emphasize the importance of primary care in identifying disorders at an early, undifferentiated stage.	

## Introduction

Fetal Alcohol Spectrum Disorder (FASD) is a group of disorders that occur in a child whose mother drank alcohol during pregnancy. Several types of damage are reported:

- Fetal alcohol syndrome (FAS) is called "full" when it includes growth retardation, craniofacial dysmorphia, and neurodevelopmental disorders. It is called "partial FAS" when it is a more moderate form without these three developmental abnormalities [1, 2].
- Alcohol-Related Neurodevelopmental Disorder (ARND): manifested by psycho-affective disorders, adjustment disorders related to memory problems, attention, behavior with hyperactivity, socialization with difficulties in social interactions [3, 4].
- Alcohol-Related Birth Defects (ARBD): are malformations, mainly cardiac and musculoskeletal, as a result of alcohol consumption during the first trimester of pregnancy [3, 4].

Excessive alcohol consumption almost always results in FAS. Because it is impossible to define thresholds at which it would consistently occur [5, 6], the French recommendation is "zero alcohol during pregnancy" [4, 7, 8].

FAS is a major public health problem. It is considered the leading cause of preventable non-genetic intellectual disability at birth and social maladjustment of the child: disruption of schooling, unemployment, mental health problems, or trouble with the law [9, 10].

A meta-analysis published in 2017 by Popova et al. estimated the prevalence of FAS at 0.146 per 100 persons worldwide [11]. In France, between 2006 and 2013, 0.48 per 100 births were diagnosed with FASD in the neonatal period, including 0.7 per 100 people with FAS [12]. This incidence is poorly known and probably underestimated. Among the French regions, Réunion is the one most affected by this syndrome [12].

According to the Leeuwenhorst Group, "the generalist is a medical doctor who provides personal, primary and continuing care to individuals, families and populations, regardless of age, sex or pathology. It is the synthesis of his or her functions that is unique" [13]. The general practitioner (GPs) serves as an individual's customary initial interface within their inherent milieu. This professional attends to all individuals, impartial to age, gender, or medical condition. Comprehensive in scope, they assimilate the entirety of biopsychosocial facets, encompassing the spectrum of potential hazards and ailments that might impact the individual. Additionally, they orchestrate the management of repercussions stemming from determinations made by fellow healthcare practitioners. In effectuating continuity and overseeing the trajectory of medical attention, these practitioners, through their ministrations to both individuals and families, actively contribute to the fulfillment of public health objectives [13].

Several studies, mainly quantitative using questionnaires, have consistently shown that general practitioners (GPs) are reluctant to address the issue of alcohol consumption during pregnancy [14–16]. The reasons were multiple: lack of time, lack of knowledge about the syndrome due to lack of training, difficulty in diagnosis due to the great variability of symptoms, difficulty in discussing alcohol with patients for fear of being stigmatized [14–16].

Réunion, located in the Indian Ocean, is a French overseas territory with a diverse and multicultural population. The region faces significant challenges related to socioeconomic precariousness and illiteracy rates. The local culture is deeply intertwined with secular knowledge, mainly centered around the cultivation of sugar cane and the consumption of rum. Notably, alcohol taxation is low and alcohol advertising is prevalent in the area [17].

Previous research has mainly focused on the detection capabilities of different health care disciplines, such as pediatricians, midwives, and others, with regard to patients with FASD. However, limited attention has been paid to the challenges faced by GPs in identifying individuals with FASD, despite their privileged position in monitoring families, which provides a unique opportunity for early identification of neurodevelopmental disorders.

The responsibilities incumbent upon family physicians situate them at the vanguard of primary prevention endeavors. Their pivotal roles encompass the discernment of indicative manifestations conducive to averting or eradicating the etiological underpinnings of health maladies, both for individual patients and broader demographic cohorts. In this context, their purview extends to the identification of behavioral patterns among women of reproductive age concerning alcohol consumption, while concurrently encompassing the early identification of prodromal cues indicative of neurodevelopmental disorders. The latter holds significance as timely recognition thereof holds the potential for accurate diagnosis and the institution of pertinent therapeutic interventions.

In 2015, the French government designated La Réunion as a pilot region for the implementation of a program to prevent and treat fetal alcohol spectrum disorders. Between 2016 and 2018, an action plan for the prevention of FAS was implemented. Six axes have been defined, including one on professional training and another on the implementation of a care pathway for affected women and children [18].

During the first evaluation of this action plan in 2019, the Regional Health Agency of La Réunion highlighted the lack of uptake by GPs in La Réunion of the training courses offered on FASD, as well as the lack of effectiveness of measures to identify alcohol consumption in pregnant women [19].

General practitioners reported difficulties in discussing alcohol during consultations, especially with women. Alcohol remains a taboo subject in French society [20].

The aim of the present study was to better understand the difficulties experienced by GPs in identifying pregnant women who drink alcohol and in identifying individuals with FASD in the pediatric or adult period. The results of the study could allow the design and implementation of interventions to overcome these difficulties and improve the identification of FASD.

## Materials and methods

### Study design and setting

The study was conducted in La Réunion, a French region where the prevalence of FASD is considered to be the highest in the country.

Two studies were conducted jointly by two researchers on the same sample of GPs. The first, conducted by François Baelen (FB), aimed to understand the barriers and motivations of general practitioners in La Réunion in the prevention of FASD, while the second, conducted by Louise Delfarguiel (LD), focused on the detection of alcohol consumption in pregnant women and the identification of individuals with FASD. This study followed a cross-sectional qualitative design, guided by the principle of grounded theory. This research approach was chosen to comprehensively explore the perceptions of GPs regarding the identification of FASD. By adopting this methodology, the study aimed to uncover barriers and motivations in an unbiased manner, allowing for a thorough examination of the topic without influencing the responses provided by the participants.

### Sampling procedure

Purposive sampling was theoretically constructed and adapted as the study progressed to recruit a diverse population of physicians with respect to age, gender, years in practice, type and mode of practice, and location (east, west, north, and south of the region). Each physician was contacted individually by telephone or e-mail. The study was introduced by the researchers (LD, FB) by mentioning the research topic without revealing the research question. Participation was completely voluntary. Informed consent was obtained. Participants were assured of anonymity. Only volunteers were selected to participate in the study. The criteria for non-inclusion were refusal to participate and GPs with a particular type of practice or practicing exclusively unscheduled care.

### Interview procedure

Two frameworks of open-ended questions were developed by the two researchers. They were then modified according to the emergence of concepts and the construction of theory (Appendix n°1). The interview framework was tested with three independent physicians.

Data were collected through individual, face-to-face, semi-structured interviews at the physicians' workplaces or via videoconference recorded by the two researchers. Informed consent was obtained from the physician at the beginning of each interview. The interviews were conducted only in French. The interviews began with the collection of demographic data: age, sex, number of years in practice, etc. The personal data (surname, first name, etc.) of the GPs were not recorded in order to limit the risk of identification. Interviews were numbered and transcribed anonymously. All audiovisual and voice recordings were destroyed at the end of the transcription process.

### Data analysis

The analysis followed the principle of anchored theorization. The interviews were transcribed verbatim and coded using Nvivo® version 11.3 software, first in an open-ended manner and then gradually organized into axial coding. As the analysis progressed, a categorization of these codings revealed concepts that were related. Theoretical saturation of the data was sought through additional interviews. The analysis of the interviews was carried out by four researchers involved in the data collection (LD, FB), and then the results were also analyzed by two other researchers (SL and MS) to ensure the triangulation of the data.

### Ethics

An anonymization step was performed during the data processing in order to avoid any personal data collection. In accordance with current regulations, a request for compliance with the reference method (MR004) was submitted to the Commission nationale de l'informatique et des libertés (CNIL), registered under number 2219132v0.

## The results

Initially, 31 GPs were contacted to participate in this study. In the end, 20 interviews were recruited. Of the remaining 11 GPs, some did not respond due to lack of time and interest in the subject, others responded positively but further data collection was no longer necessary because the theoretical saturation of data had been reached (Table 1).

**Table 1:** Characteristics of the included population of general practitioners

	**Gender**	**Age years**	**Years of practice**	**Place of exercise**	**Duration of interview minutes**	**Place of interview**
GP 1	M	30	2	Saline les Hauts	21	Medical office
GP 2	M	30	2	Saint Paul	26	Medical office
GP 3	M	60	30	Les Avirons	22	Medical office
GP 4	F	35	3	Sainte Marie	18	Video conferencing
GP 5	M	62	31	Saint Denis	32	Medical office
GP 6	F	47	17	Grand Bois	18	Medical office
GP 7	F	41	12	Saint Pierre	15	Medical office
GP 8	F	55	22	Piton St Leu	26	Medical office
GP 9	F	64	39	Saint Benoit	38	Video conferencing
GP 10	M	46	17	Salazie	26	Video conferencing
GP 11	M	64	35	Saint Joseph	12	Medical office
GP 12	M	55	26	Tampon	13	Medical office
GP 13	F	47	19	Le Port	13	Medical office
GP 14	M	67	36	Le Port	18	Medical office
GP 15	M	31	4	Saint Pierre	20	Medical office
GP 16	F	45	17	Etang Sale	16	Medical office
GP 17	M	59	30	Saint Joseph	26	Medical office
GP 18	F	38	10	Saint Joseph	16	Medical office
GP 19	M	42	15	Saint Joseph	23	Medical office
GP 20	F	39	9	Etang Sale	17	Medical office

*F* Female, *M* Man, *Video conferencing* Remote video recording

On average, the interviews lasted 21 minutes (min-max = 12-38) for 9 women and 11 men. The mean age of the GPs at the time of the interviews was 44.7 years (min=30; max=67), with a mean number of years in practice of 18.8 years (min=2; max=39).

Interviews were conducted until theoretical data saturation occurred at the 15th interview. The last 5 interviews were conducted to verify the absence of new data useful for theory construction. The data or results analyzed were not shared with the participants.

General practitioners (GPs) face numerous and diverse barriers to identifying pregnant women who consume alcohol and individuals with FASD in the pediatric and adult stages. These obstacles stem from both intrinsic factors related to the GP profession and extrinsic factors related to the societal context, particularly in Réunion. Intrinsic barriers are related to the challenges inherent in the role of the general practitioner, while extrinsic barriers are related to the prevailing taboo surrounding alcohol, especially for pregnant women, and to the social characteristics specific to Reunion.

### Taboo, denial and paradoxical social injunctions

The idea that alcohol is taboo in society, especially among women, led to the patients' denial of its use: "*I think that women don't talk about it too much...it's true that uh...we feel that it's more taboo for women than for men*." (GP 15). A denial by some GPs who, although aware of their prejudices, said they were less likely to broach the subject and underestimated alcohol consumption in this population: "*It is said that alcoholism is more hidden, more secretive among women, that they talk less about it, and it is true that, as a result, it is a problem that I broach even less often with women*" (GP 19).

GPs also expressed a sense of powerlessness in the face of paradoxical social injunctions regarding alcohol advertising in Réunion: "*There are more advertisements for rum than for FAS...*" (GP 1). Nevertheless, the prevention campaigns were considered effective by the doctors and led to a good awareness of the population of the dangers of alcohol in general: "*We have the impression that there is still better knowledge on the part of the general public.*" (GP 11).

Overall, GPs felt that, thanks to mass prevention campaigns, patients were well aware of the importance of not drinking alcohol during pregnancy: "*I think that today the majority of women know that they shouldn't drink alcohol during pregnancy...well, there are a lot of messages that are being spread lately...on the radio, also on billboards...*" (GP 7).

Alcohol and tobacco were often discussed at the first consultation at the beginning of the pregnancy and then less or not at all during the follow-up: "*Often at the pregnancy discovery consultation with the prescription of beta-HCG and then afterwards we give the little booklet and there we explain the risks during pregnancy with regard to food, tobacco, alcohol*" (GP 20).

The GPs advocated abstinence from alcohol during pregnancy: "*I tell her that from now on it's zero... we have to set the bar on alcohol*" (GP 6).

### Limited knowledge and experience

Awareness varies according to the personal experience of each doctor. Many GPs considered FAS to be a syndrome rarely seen in general practice: "*Well, FAS, I don't see it... or I miss it completely...*" (GP 7). The low prevalence of FAS meant that GPs had little experience of the condition. They reported that, despite its high local incidence, they did not think about identifying it. "*Afterwards, it is not something that I tend to detect in babies, even though I do a lot of pediatrics and prevention in children*" (GP 19).

This lack of experience led to a general lack of knowledge about the signs that can identify FASD: "*Well, how can I put it? FAS is still a bit strange for me. Well, it's clear that I feel like I don't know enough about it overall...*" (GP 7). This lack of knowledge was accentuated by the great variability of the non-specific symptoms that make up the condition: "*After that, it's probably more complex...there are different forms and it's not 1 or 0. It's polymorphic.*" (GP 3).

Overall, GPs reported a lack of training in this area: "*I don't feel that I am particularly trained now*" (GP 14).

Overall, they lamented a gradual decline in antenatal care as it became the domain of midwives and gynecologists. GPs are consulted by patients for acute pathological episodes: "*Pregnant women, it's true, we don't see them that much. We see them from time to time ... yes, when they are sick ... well, often it is rather the gynecologists who follow them*" (GP 16).

### Highly variable symptoms and care pathway

The GPs haven’t reported an impression of being in the first line for diagnosing patients with FASD. They remained uncertain about the pathway of care that lead to the diagnosis. Diagnosing FAS was often made by other health professionals: "*So I didn't make a diagnosis, in the sense that the children I know who have it, have always been diagnosed beforehand*" (GP 19) and "*They are examined by the pediatrician in the city or in the hospital before they come to the GP*" (GP 1).

Many had doubts about the care pathway following the diagnosis was made: "*I'm really embarrassed because ... There was REUNISAF before... and now I don't know..*." (GP 6) and "*Oh no, I don't know... maybe the children's hospital... no, I don't know, I would have to look, I don't know*" (GP 9).

### Ambiguous classification and method of diagnosis

The specificity of FASD symptoms within other neurodevelopmental disorders remained unclear for most GPs. According to the participants, physicians' goals should focus more on identifying acquisitional delays in general in order to refer the child early for a specific diagnosis and treatment: "*As a general practitioner, honestly, we're going to do our job of alerting as soon as we realize there's a psychomotor delay or whatever, but ... like anything, I don't mean FAS more than anything else*" (GP 13) or "*I'm qualified to assess the child's development, so I refer them to the specialist who will do their job of diagnosing a developmental disorder, and in the spectrum of developmental disorder there may be FAS.*" (GP 19)

### Lengthy consultations

GP related a perception of pressure of time in their day-to-day clinical time. Curative-centered practice remained the norm according to the GPs. Their meetings follow one on another at a pace leaving too little room for preventive and screening interventions. Identifying this disorder was all the more complex because of the limited time available for a medical meeting: "*It's true that when you have a consultation where you go from one to the other to ask yourself questions, you need to have time.*" (GP 11) or "it's not often easy and we don't see the children for very long..." (MG 16).

## Discussion

GPs reported that obstacles to the identification of FASD were the taboo and the paradoxical injunctions of society, the limited knowledge and experience, the non-specific and highly variable symptoms, the ambiguous classification and method of diagnosis involving the mobilization of a multidisciplinary team, and the length of consultations. The results of the study showed that GPs did not prioritize the identification of FASD in adults and expressed skepticism about the effectiveness of treatment, believing the disorder to be irreversible. In contrast, GPs were confident in identifying neurodevelopmental disorders resulting from a variety of causes, but were concerned about the long waiting times for access to specialist care.

### Taboo, denial and paradoxical social injunctions

Practicing in Réunion means taking local specificities into account. In Reunion, it is traditional to consume alcohol for social reasons (family celebrations or others), but also for therapeutic reasons (alcohol as a remedy in traditional medicine). A study carried out among Reunionese women with children suffering from FASD revealed a feeling of shame and guilt leading to a denial of their alcohol consumption. In fact, the patients reported that it took them a long time to admit that their drinking was excessive and to speak of "alcoholism" [21]. This denial among patients in La Réunion is in line with the observations of the GPs, who found it difficult to screen for addictive behavior because it was hidden by the patients themselves.

GPs highlighted another important aspect of La Réunion, namely the ubiquitous presence of alcohol advertising, which actively promotes its consumption. The GPs noted that this advertising environment plays a role in encouraging alcohol consumption among the population. The strong impact of these advertising campaigns on the population leads the general practitioners to feel that prevention is worthless [22]. This is encouraged by the low price of alcohol and the multiplication of outlets [23]. This could be an obstacle to the role of GPs in preventing the harmful effects of alcohol, as they do not feel supported by local public authorities.

### Limited knowledge and experience

An Australian study found that health professionals did not systematically prevent alcohol use during pregnancy because they believed that pregnant women knew about the harmful effects of alcohol on the fetus and had stopped drinking as a result [24]. This is consistent with the results of the present study, in which GPs believed that prevention campaigns were effective and that patients were well informed on the subject. They were also aware that they had prejudices about alcohol consumption among women in general, which led to an underestimation of alcoholism in this population. GPs found it challenging to address women's addictive behavior and had difficulty referring them to appropriate services for support. Nevertheless, GPs acknowledged the existence of validated questionnaires to assist in the diagnosis of pathological alcohol use, which they used during consultations to aid in the assessment process, such as AUDIT, T-ACE, TWEAK, CAGE, etc. [25, 26].

Participating GPs emphasized the importance of addressing alcohol use during the initial pregnancy consultation. This particular moment was identified as a critical opportunity to provide lifestyle advice and guidance, prompting GPs to actively inquire about alcohol use at this early stage of care. Many brief alcohol interventions for pregnant women are effective in increasing abstinence during pregnancy and preventing FASD, but even a single question about alcohol use during pregnancy can be powerful [27]. They were able to use the pregnancy booklets, which mention alcohol consumption [28]. Overall, they supported the "zero alcohol during pregnancy" policy recommended in France and in many other countries, such as the United States, Canada, and Australia. On the other hand, they tended not to ask the question again during pregnancy follow-up, for various reasons, including the fact that follow-up is increasingly carried out by other professionals, such as midwives or gynecologists. GPs saw patients mainly for acute illness and not for follow-up [14, 24].

Consistent with previous research, studies to date have reached a consensus on the central role of GPs in the prevention and detection of FASD. These studies have recognized and supported the significant contribution of GPs in both preventing cases of FASD through appropriate counseling and education, and in identifying individuals at risk or already affected by the disorder [29, 30]. However, very few GPs reported having seen patients with these disorders during their careers. As a result, they did not feel competent to deal with this disorder due to lack of experience [13, 15, 24]. They requested more training on the subject [31]. This lack of knowledge about FAS was also described by the GPs participating in the study, in fact, during the interviews they always used the acronym "FAS" and not "FASD" to describe Fetal Alcohol Spectrum Disorder. They also did not mention ARBD and ARND. This lack of precision in terminology may reflect a lack of information or the detrimental effect of the lack of consensus on the precise nomenclature of this disorder in nosographic classifications [32]. The DSM-V proposes: "Neurobehavioral Disorder Associated with Prenatal Alcohol Exposure (ND-PAE)" [33]. The ICD-10 uses the code Q86.0: "FAS (dysmorphic)" [34] without further precision.

### Nonspecific and highly variable symptoms

Healthcare professionals have difficulties in identifying FASD because of the wide variety of symptoms, the lack of standardized diagnostic tools, and the limited time available for consultation [15, 24, 35, 36].

A study comparing 5 diagnostic methods [36–39] concluded that none was better than the others for diagnosing FASD versus other neurodevelopmental disorders, but also among FAS, ARND, and ARBD, and that it would be interesting to create a standardized diagnostic method to have a single reference [35, 39].

### Lack of consensus on classification and diagnostic method

Several attempts at classification have been proposed to assess the severity of the effects of alcohol on the fetus and to make a diagnosis: Dehaene proposed FAS type I, II, III, IV according to the severity of dysmorphic damage and the knowledge of prenatal alcohol exposure [40]. The 1996 Institute of Medicine classification [35, 37, 40] refers to FAS with or without maternal alcohol exposure, partial FAS, ARBD, ARND; a revision of this classification was published in 2005 and 2016, which is more precise in diagnosis and easier to apply clinically [32]. This non-exhaustive list of terms surrounding the acronym "FAS" helps us to understand the difficulty of GPs in identifying this disorder.

The lack of knowledge of local specialized referral health care providers for patients diagnosed with FASD has been reported in the present study as well as in previous studies [12, 15]. The availability of specialized units included in the care pathway is recognized as an obstacle to the identification of FASD. However, GPs in Réunion could seek advice from the Coordination and Orientation Platform for Neurodevelopmental Disorders (PCO) [40], the FASD Resource Centre [41] or specialized associations for the orientation of their patients, but they were not aware of their existence.

It is even more difficult to improve the prognosis if the diagnosis is made late, for example in adulthood. In addition, GPs in this study rarely mentioned adults with FASD and more often spoke about identification in the pediatric population, suggesting that the prevalence of undiagnosed FASD is underestimated [42, 43]. FASD may be responsible for subsequent secondary disabilities such as disruptions in schooling, unemployment, mental health problems, trouble with the law, inappropriate sexual behavior, addictions, etc. Even if treatment is less effective because it is delayed, a diagnosis of FASD made in adulthood would make it possible to obtain support (human, financial, legal, etc.), improve quality of life and, consequently, improve prognosis [44]. It would be important to make GPs aware of the importance of identifying FASD, including in adulthood.

GPs agreed that their role was more important in identifying and referring children with FASD in general than in FASD itself. In fact, they objected that FASD is difficult to diagnose in practice for various reasons already discussed: time constraints, knowledge, or the need for a multidisciplinary team. Rare GPs feel confident in recognizing and referring children with FASD [14]. In addition, the 20 obligatory examinations of the child are fully reimbursed by the health insurance and the use of the health booklet could be an aid in identifying the delay in acquisition.

#### Lengthy consultations

The amount of time spent on prevention in the consultations of French general practitioners remains low compared with the time spent on curative care. The results of the study Pelletier-Fleury N and Al suggest that 50% of GPs report little or no primary prevention activity in almost 75% of their daily consultations [45].

The average consultation time for a French GP is 17 minutes [46]. This is not optimal for the identification of FASD in infants, children and adolescents. Alcohol consumption by women of childbearing age who wish to become pregnant could be investigated using screening tools such as AUDIT, T-ACE, TWEAK, CAGE, etc. [25, 26].

#### Strengths and limitations of the study

The strength of this study is the focus on GPs’ interpretation of their clinical role in identifying patients with FASD. We interviewed patients from a wide range of the French region with the highest prevalence of FAS, with a higher probability for GPs to meet patients with FASD.

Among the limitations of the study, three of the interviews were conducted at the physicians' place of work between consultations or at the end of the day, which may have had an influence on the length and quality of the interviews. Finally, the interviewed population could sometimes have answered a little less spontaneous and more thoughtful. A more relaxed attitude was regularly observed at the end of the recording with some-times more spontaneous discussions. Furthermore, it is noteworthy that more than one-third of the contacted GPs expressed disinterest in participating in the study. This potential sampling bias raises the concern that the GPs who did participate may have had a greater interest or motivation in identifying alcohol consumption in pregnant women or FASD. As a result, the findings should be interpreted considering this potential bias, which may limit the generalizability of the study's results to the broader GP population.

### Implications for practice and research

The study has highlighted several potential perspectives for practice based on its findings, which include:

- Providing training to general practitioners (GPs) in early identification and intervention for alcohol consumption in both the general population and specifically among pregnant women (for e.g. asking even a single question about alcohol use during pregnancy).
- Focusing on the identification and coordination of care pathways for children affected by neurodevelopmental disorders (NDDs) such as Fetal Alcohol Spectrum Disorders (FASD).
- Actively identifying and referring adults suspected of having FASD for further evaluation and support.
- Implementing educational initiatives targeting young women in middle and high school to raise awareness about the risks associated with alcohol consumption during pregnancy.

Furthermore, future studies are recommended to consider patients' perspectives on the role of GPs in identifying FASD or other NDDs. It is also suggested to conduct consensus-building approaches to develop a standardized assessment tool for diagnosing FASD in Réunion, ensuring consistency and reliability in the diagnostic process. These recommendations aim to improve the identification, intervention, and support provided by GPs for individuals affected by FASD and other NDDs.

## Conclusions

Reunion is the French region with the highest documented prevalence of FASD. However, the GPs reported difficulties in identifying patients, due to a lack of knowledge and experience, but also due to the difficulty of diagnosis with a great variability and lack of specificity of FASD symptoms, and the absence of a consensus on the classification or diagnosis of FASD. They reported a lack of knowledge about caregiving strategies and local providers. It is highly important to limit alcohol advertising, reinforce prevention messages about alcohol use among pregnant women and to have a well-identified care pathway to which GPs could refer their patients with NDD in order to benefit from an early functional assessment and care.

### Supplementary Information

**Additional file 1: Appendix.**

## Data Availability

The datasets used and/or analyzed in the current study are available from the corresponding author upon reasonable request.
