# Supplementary material for: The role of general practitioners in Reunion in detecting alcohol use in pregnant women and identifying fetal alcohol spectrum disorder: a qualitative study
Source: Arch Public Health. 2023 Dec 6;81:210. doi: 10.1186/s13690-023-01221-0 (PMC10699023; doi:10.1186/s13690-023-01221-0)
Supplement: Supplementary file 1 — Additional file 1: Appendix. [file 13690_2023_1221_MOESM1_ESM.docx]

Appendix n°1: frameworks

Ice-breaker question

- Can you tell me about the last consultation in which you were involved in addiction prevention?

Or

- Can you tell me about the last pregnancy consultation you attended?

Themes:

- In general, how do you deal with the issue of alcohol consumption in the general population?

- If you do drink, what do you do in practice?

- Do you feel comfortable managing alcohol withdrawal?

- Do you have a different approach for men and women? Depending on the answer: would alcohol be more accepted in men? Guilt more frequent in women?

- What information would you give a pregnant woman during her pregnancy?

- At the start of the pregnancy? During follow-up?

- Do you find it difficult to talk to a pregnant woman about alcohol?

- Do you monitor many pregnancies? If not, why not?

- Are patients aware of the harmful effects of alcohol on pregnancy?

- What do you think about alcohol consumption by pregnant women or women of childbearing age?

- Have you ever had to deal with a patient with FAS?

- What is your approach to spotting the consequences of foetal alcohol syndrome?

- Can you give me any signs that might suggest FAS?

- If not, why not?

- What do you do if you suspect FAS?

Have you ever had training on the subject of FAS?

Do you think GPs are well trained on the subject?

How do you think we could improve alcohol prevention among pregnant women and the detection of FAS?
